# Development and characterization of 23 polymorphic microsatellite loci for *Amentotaxus argotaenia* (Taxaceae), a relict vulnerable species

**DOI:** 10.1002/aps3.1149

**Published:** 2018-05-23

**Authors:** Qiqi Huang, Zhen Wang, Ting Wang, Yingjuan Su

**Affiliations:** ^1^ School of Life Sciences Sun Yat‐sen University Guangzhou 510275 People’s Republic of China; ^2^ College of Life Sciences Nanjing Agricultural University Nanjing 210095 People’s Republic of China; ^3^ College of Life Sciences South China Agricultural University Guangzhou 510642 People’s Republic of China; ^4^ Research Institute of Sun Yat‐Sen University in Shenzhen Shenzhen 5108057 People’s Republic of China

**Keywords:** *Amentotaxus argotaenia*, *Amentotaxus yunnanensis*, genetic variation, population structure, simple sequence repeat (SSR) marker, Taxaceae

## Abstract

**Premise of the Study:**

New microsatellite markers were developed for the vulnerable conifer species *Amentotaxus argotaenia* (Taxaceae) to investigate population genetic variation and the effects of environmental heterogeneity on genetic structure.

**Methods and Results:**

A total of 27 microsatellite loci were developed from *A. argotaenia* through a Fast Isolation by AFLP of Sequences COntaining repeats (FIASCO) protocol, of which 23 were polymorphic. These markers yielded 1–13 alleles and 1.0–7.9 effective alleles per locus; levels of observed and expected heterozygosity varied from 0.000–1.000 and 0.000–0.873, respectively. In total, 18 of the markers were transferable to the related species *A. yunnanensis*.

**Conclusions:**

These polymorphic markers are a valuable genetic resource for investigating population genetic variation and the potential for local adaptation in *A. argotaenia*.

*Amentotaxus argotaenia* (Hance) Pilg. (Taxaceae) is a dioecious evergreen conifer species that grows to 7 m in height. It has the widest distribution among all *Amentotaxus* Pilg. species and is found in southern and central China, northern Vietnam, and Laos (Farjon and Filer, 2013). *Amentotaxus argotaenia* grows on limestone, sandstone, shale, and granite and is found on steep cliffs, in ravines, and in mountain forests along the banks of streams. It prefers moist, shaded environments and exists at altitudes of 300–1100 m (Fu et al., 1999; Lin et al., 2007; Farjon and Filer, 2013). *Amentotaxus argotaenia* has a long evolutionary history, dating back to the Upper Miocene (Farjon and Filer, 2013). Populations of *A. argotaenia* have been in continual decline due to forest clearing. Hilton‐Taylor et al. (2013) have estimated that the range of *A. argotaenia* has been reduced by 20–29%, and remaining populations are small and isolated. Consequently, the species is categorized as vulnerable in China and as “Near Threatened” in the IUCN Red List of Threatened Species (Hilton‐Taylor et al., 2013). For these reasons, *A. argotaenia* is ideal for studying the effects of environmental heterogeneity on population genetic structure.

Ho et al. (2012) developed 15 polymorphic simple sequence repeat (SSR) markers in *A. formosana* H. L. Li (Taxaceae) and showed that 10 of those markers could also be amplified in *A. argotaenia*. However, when we used these SSR loci to examine population genetic variation in *A. argotaenia*, we found their amplification efficiency to be low. We developed a new set of polymorphic SSR markers for *A. argotaenia* with high amplification efficiency.

## METHODS AND RESULTS

We sampled 56 individuals of *A. argotaenia* from four natural populations in China (Appendix 1). If the population size was less than 15, all individuals were collected. To test the amplification of markers across species, we collected individual plants from one population of *A. yunnanensis* H. L. Li (Appendix 1). Genomic DNA was extracted using the modified cetyltrimethylammonium bromide (CTAB) method of Su et al. (2005).

SSR markers were developed in *A. argotaenia* using the Fast Isolation by AFLP of Sequences COntaining repeats (FIASCO) protocol for separating microsatellite‐containing DNA fragments from genomic DNA de novo (Zane et al., 2002). A single plant from the Jiuqushui population was randomly selected for microsatellite enrichment and library construction. After digestion with the restriction enzyme *Mse*I (New England Biolabs, Ipswich, Massachusetts, USA), genomic DNA was ligated to an *Mse*I adapter nucleotide pair (5′‐TACTCAGGACTCAT‐3′ and 5′‐GACGATGAGTCCTGAG‐3′) using T4 DNA ligase. The 10‐fold diluted digestion‐ligation mixture was subsequently amplified using the adapter‐specific *Mse*I‐N primers (5′‐GATGAGTCCTGAGTAAN‐3′) with the following PCR conditions: 24 cycles at 94°C for 30 s, 53°C for 60 s, and 72°C for 60 s. The linker‐adapted fragments were enriched by hybridization to single‐stranded 5′‐biotinylated microsatellite (AC)_15_ probes based on the reaction conditions of Deng et al. (2013). The repeat‐containing DNA segments were isolated using streptavidin‐coated beads (Promega Corporation, Madison, Wisconsin, USA) as described in Li et al. (2014) and further amplified using *Mse*I‐N primers and the PCR conditions described above. This enrichment procedure was repeated once, and the purified PCR fragments, enriched for microsatellites, were ligated into the pMD18T vector (TaKaRa Biotechnology Co., Dalian, China) at 16°C for 16 h and transformed into *E. coli* DH5α competent cells by transient thermal stimulation (ice bath for 30 min, 42°C water bath for 90 s, followed by ice bath for 2 min). Recombinant positive clones were selected by blue–white screening according to Cui and Su (2015) and identified by PCR using universal M13F/M13R primers with the following conditions: initial denaturation at 94°C for 10 min; followed by 25 cycles at 94°C for 30 s, 53°C for 45 s, and 72°C for 60 s; and a final extension step at 72°C for 7 min. The positive PCR products were sequenced with primer M13^+^/M13^−^ on an ABI 3730xL sequencer (Applied Biosystems, Foster City, California, USA), and SSRs were selected using SSRHunter software version 1.3 with parameters set to more than four repetitions for di‐, tri‐, and tetranucleotide repeats (Li and Wan, 2005).

Specific SSR primers were designed using Primer Premier version 5.0 (PREMIER Biosoft International, Palo Alto, California, USA) using the following parameters: primer size of 17–25 bp, product size of 90–400 bp, GC content of 40–60%, primer melting temperature of 47–60°C, and no complementarity between primer pairs. They were used to assess all 56 *A. argotaenia* individuals for polymorphisms. PCR amplification was performed in 20‐μL total reaction volumes, consisting of 0.6 μL of genomic DNA (approximately 40 ng/μL), 2 μL of 10× PCR buffer (containing Mg^2+^), 1.6 μL of dNTPs (2.5 mM each), 0.5 μL of each forward and reverse primer (10 μM), and 1.25 units of *Taq* DNA polymerase (TaKaRa Biotechnology Co.). The PCR conditions were: initial denaturation at 94°C for 5 min; followed by 35 cycles at 94°C for 50 s, the optimal annealing temperature for each SSR (Table 1) for 50 s, and 72°C for 90 s; and a final extension step at 72°C for 10 min. Cross‐species amplification was performed in *A. yunnanensis* using the same PCR conditions. The PCR products were labeled using the fluorescent dye 5‐FAM and loaded onto an ABI 3730 DNA analyzer (Applied Biosystems) along with the GeneScan 500 LIZ Internal Size Standard (Applied Biosystems). DNA fragment analysis and genotyping were performed using GeneMarker version 1.65 (SoftGenetics, State College, Pennsylvania, USA).

**Table 1:** Characteristics of the 27 SSR loci developed for *Amentotaxus argotaenia* in this study

Locus	Primer sequences (5′–3′)^a^	Repeat motif	Allele size range (bp)	*T*_a_ (°C)	GenBank accession no.
ATA‐01	F: TATCGGAGGAAGGTAGTG	(TG)_17_	276–281	52	MF838739
	R: TCAACATCTCACCCAAGT				
ATA‐02	F: GTGTCATCTTCTTTCCATCT	(TG)_5_	278–296	50	MF838740
	R: AGGTATCCAAACTAAGGGT				
ATA‐03*	F: TAATACCCCTTTGTCTACCT	(GT)_20_	305	51	MF838741
	R: TGATAAGAAGATCGAGTCATT				
ATA‐04	F: TCCCCACTGAATGGTTGA	(TG)_5_	262–286	52	MF838742
	R: CTTGGAAAACTTGAGGAATAAA				
ATA‐05*	F: CCAAAGGGTAGAAGGTGA	(AC)_9_AT(AC)_15_	420	52	MF838743
	R: GGTAAAAGATATGATGCAATG				
ATA‐06	F: GCTCATACTAACAATACACTTTTTC	(CA)_7_	92–104	55	MF838744
	R: ATGTCTTGTATGTTTGTGTGCTTCT				
ATA‐07*	F: CTTACCCCTTCACTCTTATT	(TC)_33_TCA(CA)_25_	305	52	MF838745
	R: TTTTGCCTCCTCCACATT				
ATA‐08	F: TTGTTGACGATAAAGCATG	(CA)_46_	220–264	49	MF838746
	R: TGTCTAAATAATTCCCCACT				
ATA‐09	F: AGGGTAGGAATGTGAGCA	(TG)_6_	268–282	50	MF838747
	R: GCCAAGCCGATACAATAT				
ATA‐10	F: TCATGCTTCGATAAAATGTG	(AC)_7_	193–203	49	MF838748
	R: AAAAGAGGGGTTAGTGGGT				
ATA‐11	F: GAGTCCTGAGTAACATAGGTTTGAT	(TG)_5_	203–263	57	MF838749
	R: AACAGGGTTTTTAGTATACACGAGG				
ATA‐12	F: GCAAGATCGAATGTTTCT	(TG)_5_	147–156	47	MF838750
	R: TCTTCTCGTCCAGTCAAA				
ATA‐13	F: CGTGATAATAAATAAAGCCTTGTAA	(TG)_15_	217–237	56	MF838751
	R: TTTTGTATGTAAAGTTTCCTCAGTC				
ATA‐14	F: CTTTGATTGCGTATTTTGG	(GT)_5_	271–275	49	MF838752
	R: TAGATGGTGGCATGTCGA				
ATA‐15	F: ACACCACTAGGACACAACACACTAC	(AC)_5_	112–189	57	MF838753
	R: TTGTTCCTTATCTTATTCATCTGTG				
ATA‐16	F: CTACATAGCCAATACTCCAA	(TG)_7_	287–313	50	MF838754
	R: CTCACCCATAGTTCCATAA				
ATA‐17	F: GTAGTTGTGGCATGTTGC	(AC)_25_	103–170	52	MF838755
	R: GTTAGAAGGATGAAGAGGG				
ATA‐18	F: ATGGCTATACATGAGGACTT	(CA)_5_	182–208	49	MF838756
	R: TAGGGAATTGTAGTATGGTTGT				
ATA‐19	F: TCTTGCTATGAAGGTCTATG	(GT)_5_	131–135	48	MF838757
	R: TACTACAGGGTTTTATGGTG				
ATA‐20	F: AGGAGGAAAATAAGAGCC	(AC)_23_	204–252	50	MF838758
	R: CATGATTGTACTGGGGTAT				
ATA‐21	F: CTCACTAAGGGGAGAGGGAAAAAGA	(GA)_5_	311–362	60	MF838759
	R: CTCTAATTCTGTGTTGCAGGGGTCT				
ATA‐22	F: TCTTGTCATTTCGTGTGTCAGTT	(TG)_7_	202–225	54	MF838760
	R: CACACAATAGAAGTATGCGTGATAT				
ATA‐23	F: CATTGCGTTATTACCACACTAT	(AC)_6_	274–282	54	MF838761
	R: AGGTAGTTATCTTAGTCCTCCAT				
ATA‐24	F: GTAGAGGCCACCATATAAGACAA	(TG)_17_	204–253	56	MF838762
	R: AGGCATAAGGGAGAACTACATAAAT				
ATA‐25	F: AGAGGAAGGGGTGTAGGA	(AC)_5_	147–173	54	MF838763
	R: TGAGCAAGAGTTTTGGATT				
ATA‐26^b^	F: AACATGAGACCAAAGTTCA	(AC)_19_G(CA)_9_	133	48	MF838764
	R: CGCAATCTATTAGACGC				
ATA‐27	F: AGTTGCATGTTTTGAAGTG	(CA)_9_	382–393	50	MF838765
	R: GATAATTTATAGGATCTACCGA				

Note

*T*_a_ *=* annealing temperature.

a PCR products were labeled with 5‐FAM fluorescent dye.

b Monomorphic loci.

A total of 160 positive clones were sequenced, 92 of which contained SSR loci. After discarding the short flanking regions, we selected 27 primer pairs that generated clear and reproducible bands. In total, 23 of these exhibited polymorphism and four were monomorphic (Table 1).

GenAlEx version 6.41 (Peakall and Smouse, 2006) was used to calculate genetic parameters, including number of alleles per locus, number of effective alleles per locus, and observed and expected heterozygosity. Using the same software, we tested for deviations from Hardy–Weinberg equilibrium in each population across all loci. Null alleles were evaluated using MICRO‐CHECKER version 2.2.3 (van Oosterhout et al., 2004), and tests for linkage disequilibrium were performed using GENEPOP version 4.1.4 (Rousset, 2008). Polymorphism information content was estimated using CERVUS version 3.0.7 (Kalinowski et al., 2007).

Within populations of *A. argotaenia*, number of alleles and number of effective alleles per locus ranged from 1–13 and 1.0–7.9, respectively (Table 2). Levels of observed heterozygosity and expected heterozygosity varied from 0.000–1.000 and 0.000–0.873, respectively (Table 2). Among the 23 polymorphic SSR loci, 10, eight, six, and five markers demonstrated significant departures from Hardy–Weinberg equilibrium in the Jiuqushui, Chuanping, Wugongshan, and Qiniangshan populations, respectively (Table 2). Three loci (ATA‐11, ATA‐15, and ATA‐17) harbored null alleles, and there was significant linkage disequilibrium between loci ATA‐08 and ATA‐11. The calculated polymorphism information content values ranged from 0.395 to 0.864 (Table 2). In comparison with previous estimates for *A. formosana* (Ho et al., 2012), the level of SSR variation within *A. argotaenia* was relatively high. We also evaluated the 27 SSR markers in *A. yunnanensis* and found that 18 of them were polymorphic (Table 3).

**Table 2:** Genetic parameters of the 23 polymorphic SSR loci developed for *Amentotaxus argotaenia*.^a^

Locus	Jiuqushui population (*N* = 15)				Chuanping population (*N* = 13)				Wugongshan population (*N* = 16)				Qiniangshan population (*N* = 12)				Total (*N* = 56)				
	*A*	*A*_e_	*H*_o_	*H*_e_	*A*	*A*_e_	*H*_o_	*H*_e_	*A*	*A*_e_	*H*_o_	*H*_e_	*A*	*A*_e_	*H*_o_	*H*_e_	*A*	*A*_e_	*H*_o_	*H*_e_	PIC
ATA‐01	4	2.2	0.733	0.540	3	2.6	1.000	0.618	4	2.6	0.625	0.615	4	3.6	1.000	0.726	6	3.8	0.821	0.740	0.698
ATA‐02	5	2.8	1.000	0.638	6	3.6	1.000^b^	0.722	3	2.1	1.000	0.529	6	3.3	1.000	0.701	10	3.9	1.000	0.740	0.702
ATA‐04	5	3.3	0.933^b^	0.693	3	2.2	1.000	0.536	6	3.4	0.938	0.707	5	2.7	0.917	0.628	10	4.4	0.946	0.771	0.740
ATA‐06	6	4.1	1.000	0.758	8	5.5	1.000	0.820	2	2.0	1.000^b^	0.500	4	2.4	1.000^b^	0.576	9	3.3	1.000	0.694	0.644
ATA‐08	7	3.6	0.933	0.724	3	2.1	0.923	0.530	4	3.0	0.688^b^	0.666	6	2.8	0.917^b^	0.642	13	4.9	0.857	0.797	0.780
ATA‐09	4	2.4	0.600	0.589	4	3.2	0.846	0.689	4	2.1	0.563	0.525	2	1.9	0.833	0.486	5	2.9	0.696	0.661	0.599
ATA‐10	3	2.3	0.933^b^	0.560	2	2.0	1.000^b^	0.500	2	2.0	1.000^b^	0.500	3	2.2	1.000	0.538	4	2.1	0.982	0.526	0.413
ATA‐11	5	3.5	0.867^b^	0.718	4	3.2	0.692^b^	0.689	6	3.7	0.250^b^, ^c^	0.730	3	2.1	0.917	0.531	8	4.4	0.661	0.769	0.733
ATA‐12	2	2.0	0.933^b^	0.498	2	2.0	0.923	0.497	2	1.9	0.813	0.482	3	2.2	0.667	0.542	3	2.0	0.839	0.507	0.395
ATA‐13	4	1.8	0.533	0.433	5	4.0	0.923	0.749	4	2.8	1.000	0.637	7	5.5	0.833	0.819	14	5.7	0.821	0.823	0.809
ATA‐14	2	2.0	1.000^b^	0.500	4	2.5	0.846	0.601	2	2.0	1.000^b^	0.500	4	3.6	1.000	0.719	5	2.4	0.964	0.589	0.504
ATA‐15	12	5.3	0.467^b^, ^c^	0.811	6	3.3	0.692	0.692	2	1.8	0.688	0.451	6	2.8	0.667^b^	0.646	17	4.4	0.625	0.772	0.741
ATA‐16	7	3.9	1.000	0.747	6	2.7	1.000^b^	0.633	11	7.5	0.750	0.867	6	3.7	0.917	0.733	14	5.0	0.911	0.800	0.778
ATA‐17	8	4.1	0.800^b^	0.758	9	5.5	0.769^b^	0.817	11	7.5	0.625^c^	0.867	1	1.0	0.000	0.000	18	4.9	0.571	0.797	0.777
ATA‐18	7	3.2	0.733	0.689	11	7.9	0.769	0.873	7	3.8	0.875	0.734	6	3.6	0.833	0.726	11	4.8	0.804	0.791	0.773
ATA‐19	4	3.2	0.867	0.684	3	2.2	0.615	0.544	4	2.3	0.688	0.561	4	3.0	0.583	0.670	5	2.8	0.696	0.641	0.588
ATA‐20	7	4.5	0.733^b^	0.778	8	3.7	0.615^b^	0.728	2	2.0	0.875	0.492	8	5.2	0.917^b^	0.809	19	8.0	0.786	0.876	0.864
ATA‐21	13	6.0	0.933	0.833	7	5.1	0.923	0.805	8	4.3	1.000	0.768	8	4.8	1.000	0.792	20	5.8	0.964	0.827	0.810
ATA‐22	10	5.6	1.000	0.822	8	4.8	1.000	0.793	7	4.2	0.875	0.764	4	3.0	1.000	0.670	12	5.3	0.964	0.810	0.789
ATA‐23	4	3.1	0.933	0.682	4	2.3	1.000	0.568	4	3.3	1.000	0.697	4	3.4	1.000	0.705	6	3.3	0.982	0.700	0.644
ATA‐24	2	2.0	1.000^b^	0.500	4	2.3	0.923^b^	0.568	6	2.3	0.688^b^	0.564	9	5.9	0.750^b^	0.830	15	7.6	0.839	0.869	0.855
ATA‐25	7	5.0	0.933	0.800	7	4.2	1.000	0.760	9	5.5	1.000	0.818	4	2.7	1.000	0.632	12	5.2	0.982	0.809	0.784
ATA‐27	5	4.5	1.000^b^	0.776	5	3.8	1.000^b^	0.737	9	6.9	1.000	0.855	7	3.9	1.000	0.747	11	7.2	1.000	0.860	0.845

Note

*A* = number of alleles per locus; *A*_e_ = number of effective alleles per locus; *H*_e_ = expected heterozygosity; *H*_o_ = observed heterozygosity; *N* = number of individuals sampled; PIC = polymorphism information content.

a Voucher and locality information are provided in Appendix 1.

b Significant deviation from Hardy–Weinberg equilibrium (*P* < 0.001).

c Significant possibility of presence of null alleles detected by MICRO‐CHECKER (van Oosterhout et al., 2004).

**Table 3:** Genetic parameters of 18 polymorphic SSR loci in the Malipo population (*N* = 23) of *Amentotaxus yunnanensis*.^a^

Locus	*A*	*A*_e_	*H*_o_	*H*_e_	PIC
ATA‐01	7	3.3	0.957^b^	0.700	0.647
ATA‐02	5	4.1	0.957	0.753	0.718
ATA‐04	6	2.7	0.913	0.634	0.574
ATA‐06	4	3.8	1.000^b^	0.738	0.690
ATA‐09	4	2.3	0.913^b^	0.560	0.463
ATA‐10	7	3.0	0.913^b^	0.664	0.605
ATA‐11	7	4.0	0.783^b^	0.748	0.716
ATA‐12	5	2.6	1.000	0.610	0.533
ATA‐13	9	2.9	0.609	0.659	0.630
ATA‐15	7	5.2	0.913^b^	0.806	0.778
ATA‐16	5	2.7	0.957^b^	0.632	0.562
ATA‐18	6	3.3	0.870^b^	0.696	0.648
ATA‐19	4	3.4	0.957	0.710	0.657
ATA‐20	3	2.3	1.000^b^	0.572	0.480
ATA‐22	12	8.0	1.000	0.875	0.862
ATA‐23	5	3.3	0.870	0.697	0.643
ATA‐24	13	6.1	1.000^b^	0.836	0.818
ATA‐25	6	3.6	0.870^b^	0.726	0.684

Note

*A* = number of alleles per locus; *A*_e_ = number of effective alleles per locus; *H*_e_ = expected heterozygosity; *H*_o_ = observed heterozygosity; *N* = number of individuals sampled; PIC = polymorphism information content.

a Voucher and locality information are provided in Appendix 1.

b Significant deviation from Hardy–Weinberg equilibrium (*P* < 0.001).

## CONCLUSIONS

We developed 23 new polymorphic SSR markers for *A. argotaenia*, 18 of which were successfully amplified in the congener *A. yunnanensis*. These SSR markers are a valuable genetic resource and may be used to evaluate overall population genetic structure, to quantify genetic variation, and to assess the potential for adaptation in *A. argotaenia*.

## APPENDIX 1

### Voucher and location information for the species and populations used in this study. Voucher specimens have been deposited in the Herbarium at Sun Yat‐sen University (SYS), Guangzhou, Guangdong, China.

**Table:**

Species	Population (Population code)	Voucher no.	Collection locality	Geographic coordinates	*N*
*Amentotaxus argotaenia* (Hance) Pilg.	Jiuqushui (JQS)	LXP134282	Jiuqushui, Hunan, China	26°34′02.10″N, 114°04′42.27″E	15
	Chuanping (CP)	LXP1307901	Chuanping, Jiangxi, China	26°45′26.36″N, 114°10′12.63″E	13
	Wugongshan (WGS)	WGS1327	Wugongshan, Jiangxi, China	27°27′53.10″N, 114°09′56.63″E	16
	Qiniangshan (QNS)	SZ12264	Qiniangshan, Guangdong, China	22°31′31.52″N, 114°32′27.37″E	12
*A*. *yunnanensis* H. L. Li	Malipo (MLP)	MLP199803	Malipo, Yunnan, China	23°07′10.25″N, 104°50′29.48″E	23

Note

*N* = number of samples.
